# The influence of the winter North Atlantic Oscillation index on hospital admissions through diseases of the circulatory system in Lisbon, Portugal

**DOI:** 10.1007/s00484-016-1214-z

**Published:** 2016-07-27

**Authors:** Ricardo Almendra, Paula Santana, João Vasconcelos, Giovani Silva, Fábio Gonçalves, Tércio Ambrizzi

**Affiliations:** 1 Centre of Studies on Geography and Spatial Planning, University of Coimbra, Coimbra, Portugal; 2 School of Tourism and Maritime Technology, Polytechnic Institute of Leiria, Leiria, Portugal; 3 CEAUL and Department of Mathematics-IST, Universidade de Lisboa, Lisboa, Portugal; 4 Atmospheric Sciences, University of Sao Paulo, Sao Paulo, Brasil

**Keywords:** North Atlantic Oscillation, Circulatory system diseases, Air pollution, Hospital admissions

## Abstract

The aim of this paper is to analyze the relationship between North Atlantic Oscillation (NAO), meteorological variables, air pollutants, and hospital admissions due to diseases of circulatory systems in Lisbon (Portugal) during winter months (2003–2012). This paper is one of the few studies analyzing the impact of NAO on health through its influence on thermal stress and air pollution and is the first to be conducted in Lisbon. This study uses meteorological data (synthetized into a thermal comfort index), air pollutant metrics, and the NAO index (all clustered in 10-day cycles to overcome daily variability of the NAO index). The relationship between morbidity, thermal comfort index, NAO index, and air pollutants was explored through several linear models adjusted to seasonality through a periodic function. The possible indirect effect between the NAO index and hospital admissions was tested, assuming that NAO (independent variable) is affecting hospital admissions (outcome variable) through thermal discomfort and/or pollution levels (tested as individual mediators). This test was conducted through causal mediation analysis and adjusted for seasonal variation. The results from this study suggest a possible indirect relationship between NAO index and hospital admissions. Although NAO is not significantly associated with hospital admissions, it is significantly associated with CO, PM_2.5_, NO, and SO_2_ levels, which in turn increase the probability of hospitalization. The discomfort index (built with temperature and relative humidity) is significantly associated with hospital admissions, but its variability is not explained by the NAO index. This study highlights the impacts of the atmospheric circulation patterns on health. Furthermore, understanding the influence of the atmospheric circulation patterns can support the improvement of the existing contingency plans.

## Introduction

Environmental health determinants such as atmospheric pollution or extreme temperatures are important public health concerns and significant risk factors to several diseases (Mercer 2003, Borrego et al. 2009).

In recent years, the impacts of extreme temperatures have become an important matter of concern to the epidemiology scientific community (Basu 2009). Despite the higher levels of cold-related mortality, the impacts of heat waves on health continue to attract attention from the media (Rau 2006, Berko et al. 2014). To analyze the consequences of exposure to extreme ambient temperature, several authors have synthetized biological resistance to weather conditions by calculating composite indexes (Steadman 1979, Panagiotakos et al. 2004, Monteiro et al. 2012, Vasconcelos et al. 2013) that combine metrics of multiple weather variables (Basu 2009) (i.e., temperature, relative humidity, dew point temperature, wind speed) and are, theoretically, more effective than standard meteorological metrics.

The association between mortality and temperature has been described as a nonlinear J- or U-shaped relationship (Kalkstein and Greene 1997). Exposure to low temperatures leads to several biological responses from the human body such as a higher concentration of red and white cells, platelets, cholesterol and fibrinogen, and increased blood viscosity that increases the likelihood of death (Eurowinter Group 1997, Keatinge 2002, Wilson et al. 2010). On the other hand, when the body temperature rises, blood flow generally shifts from the vital organs to underneath the skin’s surface in an effort to cool down increasing the stress of heart and lungs (Basu 2009).

In the same way, exposure to high levels of atmospheric pollution is linked with several health impacts, such as diseases of the respiratory, circulatory, immune, hematological, and neurological systems (Curtis et al. 2006). Furthermore, Peters et al. (2001) and Lee et al. (2002) found a significant statistical association between pollution and diseases of the circulatory and respiratory systems even when the concentration levels of pollutions were below the threshold established by the World Health Organization (WHO), suggesting that the air quality thresholds may not be indicative of clean air.

Additionally, the effect of air temperature and air quality on health are also related to each other as the effects of extreme temperatures are maximized by exposure to high levels of air pollution. At the same time, individuals exposed to extreme temperatures are more vulnerable to high levels of air pollution (Gordon 2003, Ren et al. 2006, Burkart et al. 2013).

Both meteorological variables and air pollutants can be affected by global-scale phenomena such as ENSO in South America and other regions or the North Atlantic Oscillation (NAO), in Europe. The NAO is one of the major modes of atmospheric circulation in the Northern Hemisphere, influencing the ecological and environmental systems, energy production and consumption, agriculture, industry, environmental conditions, and human health (Bojariu and Gimeno 2003), mainly during the winter period (McGregor 2005). The NAO index is traditionally defined as the difference of sea level pressure between the tropical Azores high and polar Icelandic low; it indicates the variability in atmospheric pressure and has two phases.

Positive NAO phases are characterized by high differences between the two centers of action (Ulbrich et al. 2012) and are responsible for minimal cloud cover and dry conditions in the southern European countries (López-Moreno and Vicente-Serrano 2008) and positive temperature anomalies during the day in the Iberian Peninsula (more intense at the center of Iberian Peninsula) (Trigo et al. 2002).

Negative phases are characterized by the weakening of the gradient between the Azores and Iceland centers of action (López-Moreno and Vicente-Serrano 2008). During this phase, the westerly flow is weaker allowing weather fronts with a western path (associated with high precipitation levels) to come into the Iberian Peninsula (López-Moreno and Vicente-Serrano 2008, Ulbrich et al. 2012).

NAO controls meteorological factors that influence the transport, dispersion, and concentration of pollutants (Christoudias et al. 2012), which are both of natural and anthropic genesis. Precipitation provokes wet deposition, wind promotes transport and dispersion, while solar radiation and temperature can increase chemical and photochemical reactions (Jacob and Winner 2009, Jerez et al. 2013).

Given the impacts of NAO on meteorological conditions and air quality, it should be treated as an important factor influencing several diseases. Nonetheless, the relationship between NAO and health has not yet been completely explored. Several studies assess its impact on weather conditions (Trigo et al. 2002, Hurrell et al. 2003, López-Moreno and Vicente-Serrano 2008) and on air quality (Creilson et al. 2003, Christoudias et al. 2012, Jerez et al. 2013); however, fewer studies address the relationship between NAO and health. In order to assess the influence of NAO on health, some authors have analyzed the direct relationship between the NAO index and morbidity or mortality (Messner et al. 2003, Hubálek 2005), while others have explored the causal relation between NAO, weather conditions, and diseases (McGregor 2005) or between NAO, air quality, and diseases (Pausata et al. 2013).

The aim of this paper is to analyze the influence of NAO on morbidity due to diseases of the circulatory system during winter months (2003–2012) in Lisbon (Portugal). The “Results” section presents the results of the relationship between: (a) NAO and morbidity; (b) thermal comfort and morbidity; (c) air quality and morbidity; and (d) NAO, thermal comfort, air quality and health.

## Data and methods

### Location

**Fig. 1:**
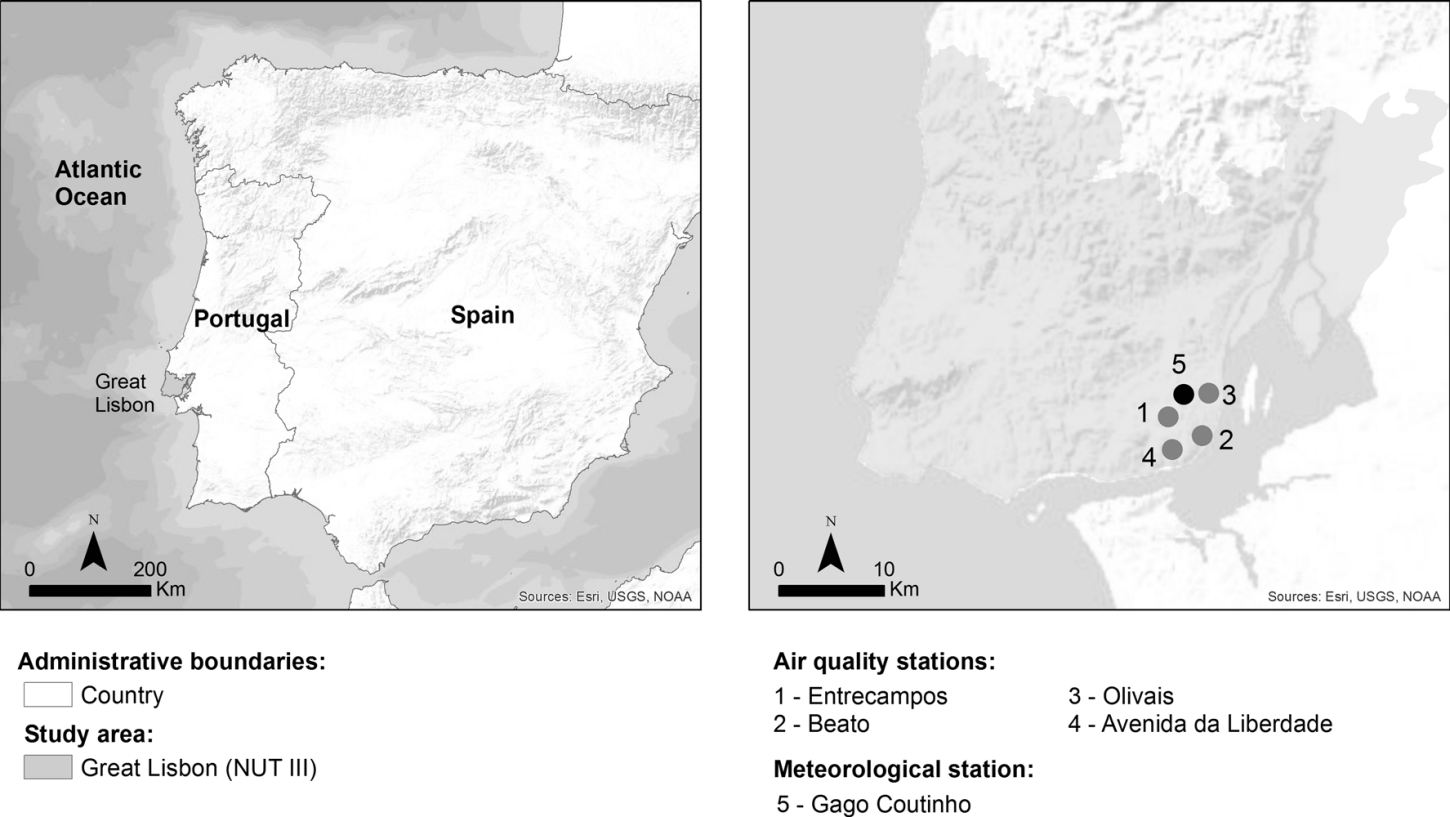
Location of the study area and selected stations

The study focused on the region (third level of the European nomenclature of territorial units for statistics) of Great Lisbon (hereafter called Lisbon) in Portugal, Iberian Peninsula (Fig. 1). Lisbon is located at 38° 42’ N and 9° 00’ W and is characterized by a rugged terrain of up to 300 m of altitude and is influenced by a typical Mediterranean climate with mild and wet winters and dry and warm summers (Csa according to the Köppen-Geiger classification). Lisbon is the most populous Portuguese region and economically the most dynamic, with about 20 % of all the country’s population (nearly 2 million) and contributing to 36 % of the gross domestic product (INE 2014).

### Data

To analyze the relationship between NAO, environmental conditions, and health impact, daily data were collected from 2003 to 2012:

- The daily NAO index was provided by the *National Oceanic and Atmospheric Administration*;
- The daily temperature (median, maximum, and minimum), precipitation, relative humidity, and wind speed were provided by *NNDC climate data online* for the Gago Coutinho meteorological station;
- Hourly concentrations (μg/m^3^) of carbon monoxide (CO), PM_10_, nitrogen dioxide (NO_2_), nitric oxide (NO), sulfur dioxide (SO_2_), PM_2.5_, and ozone (O_3_) were provided by the Portuguese Environment Agency. Four stations were selected according to the availability of data: Entrecampos (PM_2.5_), Beato (O_3_), Avenida da Liberdade (CO, PM_10_), and Olivais (NO, NO_2_, SO_2_) (Fig. 1). In case of missing values, they were estimated by calculating the average value of the nearest four stations.
- The numbers of daily urgent hospital admissions of Lisbon inhabitants due to diseases of the circulatory systems (ICD9: 390–459) were provided fully anonymized by the *Administração Central do Sistema de Saúde.*

### Methods

According to Feldstein (2000) and Bojariu and Gimeno (2003), the daily variability of the NAO index can be controlled by using cycles of 9.5 days. Thus, daily values were clustered in discrete 10-day block cycles (first cycle goes from the January 1st until the 10th, second from the 11th to the 20th and so on until the end of the year, restarting in the following year). To each cycle, several metrics were calculated: (a) average values of the thermal comfort index, wind speed, NAO index, PM_2.5_, CO, SO_2_, NO_2_, NO, O_3_ (average of the daily maximum values) and (b) the accumulated values of hospital admissions and precipitation were calculated.

Cycles starting in the winter months were selected for inclusion in the analysis. In accordance with previous works, December, January, February, and March were selected as winter months (Trigo et al. 2002, Johnson and Griffiths 2003, Almendra et al. 2012).

In order to synthetize the human vulnerability to weather conditions, four thermal comfort indexes were tested (Table 1): discomfort index (DI) according to Giles et al. (1990), effective temperature (ET) according to Ono and Kawamura (1991), effective temperature taking into account the effect of wind (ETw) according to Suping et al. (1992), and wind chill equivalent temperature (WC) according to Osczevski and Bluestein (Osczevski and Bluestein 2005). All indexes were analyzed with maximum, average, and minimum temperatures, resulting in four sets with three indexes each.

**Table 1:** Thermal comfort indexes

	DI_min_	DI	DI_max_	ET_min_	ET	ET_max_	ETw_min_	ETw	ETw_max_	WC_min_	WC	WC_max_
Formula	T−0.55 × [(1–0.1 × RH) × (T−14.5)]			T−0.4 × [(1−RH/100) × (T−10)]			37−(37−T)/(0.68–0.0014 × RH + 1/(1.76 + 1.4 × W0.75))−0.29 × T × (1−RH/100)			13.12 + 0.6215 × T−11.37 × W + 0.3965 × T × W		

*DI* discomfort index, *ET* effective temperature, *ETw* effective temperature with wind, *WC* wind chill, *T* temperature, *RH* relative humidity, *W* wind speed

The relationships between weather and morbidity were explored through linear regression models. According to Pollock (1999), in order to control for seasonal variation, the fitted models included a periodic function:

$$ y={\beta}_0+{\beta}_1 \sin \left(\left(\frac{2\pi }{P}\right)t\right)+{\beta}_2 \cos \left(\left(\frac{2\pi }{P}\right)t\right)+{\in}_t $$

where *β*_0_ is the intercept, *β*_1_ and *β*_2_ are the trigonometrical functions of frequencies, *P* corresponds to the period and *t* to the length. Several models were fitted using the R package Hmisc (http://cran.r-9project.org/web/packages/Hmisc/index.html) to explore the association between the following: (a) NAO index and hospital admissions, (b) NAO index and thermal comfort indexes, (c) thermal comfort indexes and hospital admissions, (d) NAO index and pollution levels, (e) pollution levels and hospital admissions, and (vi) pollution levels and the thermal comfort indexes. Both *β*_1_ and *β*_2_ terms were kept in the models despite their significance value in order to maintain the results adjusted for seasonal variation and to provide better comparison between the several models tested.

This study looked for possible indirect effects between the NAO index and hospital admissions, assuming that the effect of NAO (independent variable) on hospital admissions (outcome variable) can be through thermal discomfort and/or pollution levels (tested as individual mediators). In order to perform the corresponding analysis, the R-mediation package (http://cran.r-norg/web/packages/mediation/index.html) was used (Tingley et al. 2014). This takes various standard model objects, which correspond to mediator and outcome model components (both fitted for seasonal variation according to the proposed method). We used linear regression model for the mediator and outcome components and nonparametric bootstrapping (1000 resamples) for the corresponding variance estimation (Efron and Tibshirani 1994).

In regression analysis, although we usually aim to estimate casual effects of a covariate (independent variable) on an outcome (dependent variable), we can sometimes be interested in the process in which the covariate casually affects the outcome. Mediation analysis specifies the relationship between an independent variable and a dependent variable by considering a mediator variable to explaining the mechanism by which they are related. This method provides information about the average causal mediation effect (ACME) and average direct effect (ADE) representing, respectively, the effect of the independent variable on the dependent that is explained by a given set of mediators and the effect of the independent variable on the dependent variable unexplained by those same mediators. ACME is identified under specific assumption of conditional independence among outcome, mediator (pollutants), and treatment (NAO index); that assumption may be strong in many applied settings. Thus, we assessed the sensitivity of an estimated ACME to unmeasured confounding, also implemented in mediation R package, considering different values of the correlation between outcome and mediator model residuals (Imai et al. 2010, Richiardi et al. 2013).

## Results

In our study, 66,162 hospital admissions for diseases of the circulatory system were recorded during 110 cycles of 10 days each (Table 2). The mean concentration of air pollutants per cycle was 53.7, 44.0, 31.1, 29.9, 20.0, 1.7, and 69.0 μg/m3 for CO, NO_2_, PM_10_, NO, PM_2.5_, SO_2_, and O_3_, respectively. The average air temperature was 11.9 °C. The NAO index ranges between 1.6 and 1.5, with an average of 0.13.

**Table 2:** Descriptive statistics of the variables under analysis

		Mean	Minimum	Maximum	Standard deviation
Hospital admissions	Diseases of the circulatory system (*n*)	601	503	740	46.5
Meteorological conditions	Average temperature (°C)	11.9	8.0	17.0	2.0
	Maximum temperature (°C)	15.7	11.1	22.8	2.4
	Minimum temperature (°C)	8.8	4.8	12.8	2.0
	Precipitation (mm)	23.8	0.0	142.0	29.5
	Relative humidity (%)	73.8	49.7	92.0	9.4
	Wind speed (m/s)	3.2	1.9	5.2	0.7
Atmospheric circulation	NAO index	0.13	−1.6	1.5	0.7
Air pollutants	CO (μg/m3)	53.7	280.3	1198.0	191.4
	O_3_ (μg/m3)	69.0	30.5	114.2	16.2
	NO_2_ (μg/m3)	44.0	23.9	73.9	11.2
	PM_10_ (μg/m3)	31.1	13.8	61.2	10.0
	NO (μg/m3)	29.9	3.5	106.8	21.9
	PM_2.5_ (μg/m3)	20.0	8.0	50.0	9.1
	SO_2_ (μg/m3)	1.7	0.1	6.5	1.5

### North Atlantic Oscillation and hospital admissions

The association between the NAO index and hospital admissions through diseases of the circulatory system was not statistically significant (coeff.: −0.80, std. error: 6.18, *p* value: 0.89).

### Thermal comfort and hospital morbidity

Four sets of thermal indexes were calculated: WC uses temperature and wind, DI and ET are built using temperature and relative humidity, and ETw adds wind. To identify the index that best expresses vulnerability to thermal conditions, linear models associating hospital admissions and the comfort index were built (Table 3).

**Table 3:** Association between comfort indexes and hospital morbidity

Hospital admissions	Comfort indexes	DI		ET		WC		ETw	
		Coeff.	Std. error	Coeff.	Std. error	Coeff.	Std. error	Coeff.	Std. error
Circulatory system	Minimum	−7.51*	3.06	−6.17*	2.75	−4.52*	1.98	−4.42*	1.98
	Average	−7.80*	3.16	−6.85*	2.91	−5.08*	2.06	−4.30*	1.96
	Maximum	−5.94*	2.86	−5.56*	2.69	−4.07*	1.85	−3.27	1.75

*p < =0.05

The comfort indexes are significantly associated with hospital morbidity, and the coefficients tend to be higher when the discomfort indexes do not include wind. Although only winter months are being analyzed, thermal comfort indexes built with average temperature are strongly associated with hospital morbidity due to diseases of the circulatory system than those indexes using minimum temperature.

Considering the results, the DI was selected as the comfort index that best characterizes vulnerability to thermal conditions (being also the thermal comfort index with lower *p value* in the models).

### Air quality and hospital admissions

The relationship between the selected air pollutants and hospital admissions was analyzed. Excepting O_3_, all pollutants are positively associated with hospital admissions for diseases of the circulatory system and only NO_2_ is not significantly associated (Table 4).

**Table 4:** Association between air pollutants and hospital admissions

Hospital admissions	CO (μg/m3)		O_3_ (μg/m3)		PM_2.5_ (μg/m3)		NO (μg/m3)		SO_2_ (μg/m3)		NO_2_ (μg/m3)		PM_10_ (μg/m3)	
	Coeff.	Std. error	Coeff.	Std. error	Coeff.	Std. error	Coeff.	Std. error	Coeff.	Std. error	Coeff.	Std. error	Coeff.	Std. error
Circulatory system	0.12**	0.02	−1.14**	0.38	2.30**	0.44	0.71**	0.22	14.88**	2.63	0.43	0.40	0.90*	0.43

*p < =0.05; **p < =0.01

### North Atlantic Oscillation, thermal comfort, air quality, and hospital admissions

No significant association was found between the NAO index and DI (coeff.: 0.28, std. error: 0.18, *p* value: 0.12) (the comfort indexes that included wind are significantly associated with NAO in their average and maximum component, but not in the minimum). As mentioned before, the DI combines temperature and relative humidity, and none of them is significantly associated with the NAO index. In fact, both high and low NAO indexes can be related to different synoptic situations that are linked with similar temperature and relative humidity but completely different precipitation levels (Table 5).

**Table 5:** Comparison between the cycles with higher and lower NAO index under analyses

Date (month/year)	NAO index	Average temperature (°C)	Maximum temperature (°C)	Minimum temperature (°C)	Precipitation (mm)	Relative humidity (%)
01/2005	1.5	9.4	13.3	6.1	0	77.6
12/2009	−1.6	10.5	13.9	7.9	19.1	81.3

**Fig. 2:**
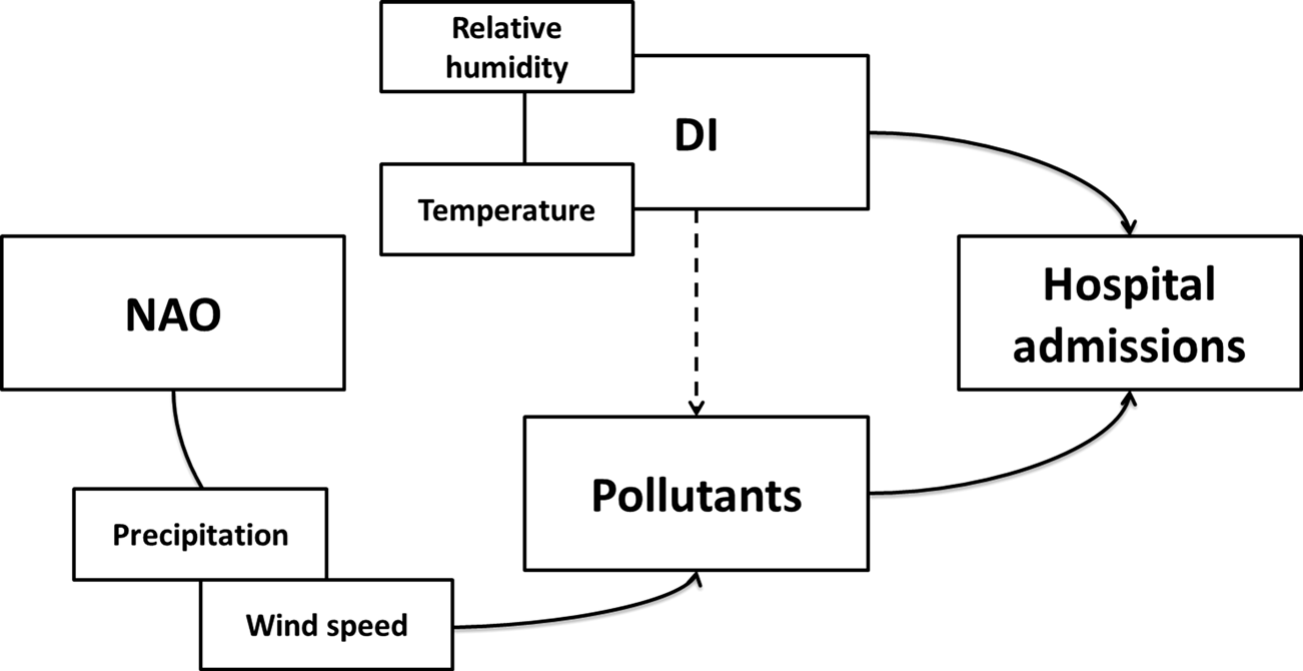
Proposed framework model of the influence of North Atlantic Oscillation on hospital admissions for circulatory diseases

NAO index is significantly associated with CO, PM_2.5_, NO, NO_2_, and PM_10_ (Table 6). Similarly, the air pollutant level significantly decreased while the wind speed and precipitation increased, except O_3_ which has a different behavior, associated with fair weather, no wind, and strong sunlight. NAO is also significantly associated with precipitation (coeff.: −17.7, std. error, 3.69, *p* value: 0.00) and wind speed (coeff.: −0.31, std. error: 0.09, *p* value: 0.00) (Fig. 2).

**Table 6:** Association between air pollutants, NAO index, precipitation, and wind speed

	CO (μg/m3)		O_3_ (μg/m3)		PM_2.5_ (μg/m3)		NO (μg/m3)		SO_2_ (μg/m3)		NO_2_ (μg/m3)		PM_10_ (μg/m3)	
	Coeff.	Std. error	Coeff.	Std. error	Coeff.	Std. error	Coeff.	Std. error	Coeff.	Std. error	Coeff.	Std. error	Coeff.	Std. error
NAO index	54.96*	23.5	0.45	1.51	2.43*	1.18	6.43*	2.53	0.34	0.20	3.65*	1.43	2.83*	1.35
Precipitation	−1.93**	0.54	0.01	0.04	−0.08**	0.03	−0.18**	0.06	−0.01*	0.00	−0.13**	0.03	−0.10**	0.03
Wind speed	−123.3**	21.18	3.71*	1.49	−6.41**	1.05	−16.96**	2.06	−0.74**	0.19	−9.98**	1.13	−7.85**	1.16

*p < =0.05; **p < =0.01

As previously mentioned, no significant linear association between the NAO index and morbidity was found. The absence of direct association was also supported by the results of the causal mediation analyses: the average direct effect and total effect of NAO index in hospital admissions are not significant. However, NAO influences air quality and, through this indirect effect, can also affect human health (Fig. 2). The estimated average causal mediation effect is significantly different from zero in some pollutants (CO, PM_2.5_, NO, and SO_2_). The results suggest that the NAO is associated with CO, PM_2.5_, NO, and SO_2_ levels, which in turn increase the probability of hospitalization due to diseases of the circulatory system. The estimated average increase in hospitalizations resulting from the indirect effect of NAO is 6.84 (*p* value <0.01) through CO, 5.86 (*p* value <0.01) through PM_2.5_, 4.92 (*p* value <0.01) through NO, and 5.27 through SO_2_ (Table 7).

**Table 7:** Summary of indirect effect of North Atlantic Oscillation over hospital admissions

Mediators	Average causal mediation effect	Average direct effect
DI	−2.26	1.45
CO	6.84**	−7.64
PM_2.5_	5.86*	−6.66
O_3_	−0.52	−0.29
NO	4.92**	−0.80
SO_2_	5.27*	−6.07
NO_2_	1.7	−2.51
PM_10_	2.68	−3.49

*p ≤ 0.05; **p ≤ 0.01

The results from sensitivity analyses supported the robustness of our findings, by checking ACME estimation for several referential NAO index values; it was possible to conclude that ACME increases as NAO index increases, and there is no change on the statistical significance of ACME and ADE.

## Discussion

This study assesses the influence of NAO on health in Lisbon (during the winter months from 2003 to 2012). The results from this analysis show a possible indirect relationship between NAO and hospital admissions from circulatory diseases. This relationship is established through the indirect effect of pollution: NAO is associated with CO, PM_2.5_, NO, and SO_2_ levels, which in turn increase the probability of hospitalization. Hospital morbidity significantly increases with thermal stress (the DI is significantly associated with hospital morbidity due to diseases of the circulatory system diseases), although, thermal stress is not explained by the NAO index. Previous studies conducted in Europe have shown the effects of NAO on health. Messner et al. (2003) found a consistent positive relation between increasing NAO index and an increase in acute myocardial mortality in Sweden. Hubálek (2005) analyzed the impact of NAO on the incidence of some infectious diseases in the Czech Republic and found significant correlations between them. McGregor (2005) found statistically significant inverse associations between mortality from ischemic heart disease and the climate index representing the interaction between the NAO and temperature across England. Pausata et al. (2013), assessing the particulate matter variability induced by NAO in Europe during winter and the potential impact on human health, found that positive shift in the mean winter NAO of one standard deviation would lead to about 5500 additional premature deaths in Mediterranean countries due to the increase in particulate matter concentration.

Nevertheless, as described above, we found significant positive linear associations between the NAO index and CO, PM_2.5_, NO, and NO_2_. Similar results were also mentioned by Jerez et al. (2013) and Christoudias et al. (2012) which reported lower concentration of pollutants in southern Europe during NAO negative phases. These results show the processes of transport and deposition of air pollutants through the effect of wind and precipitation.

Air pollutants are positively associated with hospital admissions (except O_3_ and NO_2_); previous studies analyzing the health impact of several air pollutants also reported important differences between them: Forastiere et al. (2005), in Rome, identified significant increases of out-of-hospital coronary deaths with CO and PM_10_, but not with NO_2_; Cendon et al. (2006) found stronger positive correlations between SO_2_ and daily hospitalizations for myocardial infarctions than between CO, O_3_, NO_2_, and PM_10_.

O_3_ is negatively associated with hospital admissions; however, there is no causal relationship between the decreasing levels of ozone and the hospital admission increase. This apparent protective effect results from the O_3_ increase on warm days, while the relation between hospital admissions and temperature is inverse (Moolgavkar et al. 1995, Ito et al. 2005, Medina-Ramón et al. 2006). Similar results were found in the previous research (Moolgavkar et al. 1995, Medina-Ramón et al. 2006).

The thermal comfort index is not significantly associated with the NAO index. Unlike the countries of Central and North Europe where a strong association is found between the NAO index and temperature (Osborn et al. 1999, Trigo et al. 2002, Hurrell et al. 2003), in Lisbon, this relationship was not recorded. The results of this study are supported by the study of Ulbrich et al. (2012), showing that the relationship between the NAO index and temperature is not linear in the Iberian Peninsula.

Summarizing, hospital morbidity is positively associated with the pollutant levels (except ozone and NO_2_, as reported previously) and is negatively associated with the thermal comfort index, although no significant direct association with NAO was found. A similar pattern was also found in previous studies addressing vulnerability to cold weather (Almendra et al. 2012, 2016, Vasconcelos et al. 2013) or exposure to high levels of air pollution (Borrego et al. 2009, Slezakova et al. 2011) in Portugal.

### Strengths and limitations

This study is one of the few analyzing the impact of NAO on health and is the first to be conducted in Lisbon. Considering the geographical and socioeconomic context of Portugal and the high vulnerability to harmful environmental conditions is fundamental to have a better understanding of the relationship between atmospheric conditions and health to effectively assess environmental risks. Thus, this study represents an important contribution to the current body of literature.

However, the results of this study must be interpreted with caution. Time series analyses were carried out for one location as such; the results should not be derived to other regions with different geographic and socioeconomic frameworks.

The methods applied in this study tested for direct and indirect linear associations; however, the relationship between environmental conditions and health is often studied by nonlinear modeling providing better fitting models. Therefore, the linear mediation model is an alternative to (direct) nonlinear models and can also be employed as an exploratory and complementary tool to those models.

## Conclusions

This study investigated the effects of NAO on emergency hospital admissions from diseases of the circulatory system during the winter months in Lisbon. It was found that the NAO influences human health through its impacts on atmospheric pollutants. Positive NAO phases are associated with higher levels of air pollutants. No significant association was found between NAO and the discomfort index (built with temperature and relative humidity).

Although it is not possible to extrapolate from this to other countries or other areas of Portugal, this study draws attention to the impacts of the patterns of atmospheric circulation in the North Atlantic on human health and to the vulnerability to environmental factors.

This article can provide insights to improve public health policies and alert systems. A better understanding of the relationship between the NAO and health can help improve existing contingency plans, develop more effective adaptation strategies, and ensure they are put into action in a timely manner, thereby helping to decrease the health impacts of harmful environmental conditions.
